# Biodiversity and Physiological Characteristics of Novel *Faecalibacterium prausnitzii* Strains Isolated from Human Feces

**DOI:** 10.3390/microorganisms10020297

**Published:** 2022-01-26

**Authors:** Wenbing Hu, Wenyu Gao, Zongmin Liu, Zhifeng Fang, Jianxin Zhao, Hao Zhang, Wenwei Lu, Wei Chen

**Affiliations:** 1 State Key Laboratory of Food Science and Technology, Jiangnan University, Wuxi 214122, China; hu_wenbing@163.com (W.H.); 6200113023@stu.jiangnan.edu.cn (W.G.); 7180112023@stu.jiangnan.edu (Z.L.); zhifengf@foxmail.com (Z.F.); jxzhao@jiangnan.edu.cn (J.Z.); zhanghao61@jiangnan.edu.cn (H.Z.); chenwei66@jiangnan.edu.cn (W.C.); 2 School of Food Science and Technology, Jiangnan University, Wuxi 214122, China; 3 National Engineering Research Center for Functional Food, Jiangnan University, Wuxi 214122, China; 4 (Yangzhou) Institute of Food Biotechnology, Jiangnan University, Yangzhou 225004, China; 5 Wuxi Translational Medicine Research Center and Jiangsu Translational Medicine Research Institute Wuxi Branch, Wuxi 214122, China

**Keywords:** *F. prausnitzii*, isolation, antimicrobial resistance, carbohydrate utilization, phylogenetic analysis

## Abstract

*Faecalibacterium prausnitzii* is prevalent in the human gut and is a potential candidate for next-generation probiotics (NGPs) or biotherapeutics. However, the biodiversity and physiological characteristics of *Faecalibacterium prausnitzii* remain unclear. This study isolated 26 novel *F. prausnitzii* strains from human feces using a combination of negative screening and prime-specific PCR amplification (NSPA). Based on a 16S rRNA gene analysis, *F. prausnitzii* strains can be classified into two main phylogroups (phylogroups I and II), which were further clustered into five subgroups (I-A, II-B, II-C, II-D, and II-E). The ultrastructure, colony morphology, growth performance, and short-chain fatty acids (SCFAs)-producing ability were found to be variable among these *F. prausnitzii* isolates. The optimal pH for the isolates growth ranged between 6.0 and 7.0, while most isolates were inhibited by 0.1% of bile salts. Antimicrobial resistance profiles showed that all *F. prausnitzii* isolates were susceptible to vancomycin, whereas >80% were kanamycin and gentamicin resistant. Additionally, all strains can utilize maltose, cellulose, and fructose but not xylose, sorbose, and 2′-FL. Overall, our work provides new insights into the biodiversity and physiological characteristics of *F. prausnitzii*, as well as the choices of strains suitable for NGPs.

## 1. Introduction

The human gastrointestinal tract harbors a large number of microbes that facilitate digestion and nutrient absorption, affect host metabolism, and shape immunity [1]. Although the gastrointestinal tract contains 500–1000 bacterial genera, only a few are predominant (*Bacteroides*, *Clostridium*, *Bifidobacterium*, and *Faecalibacterium*) [2]. The genus *Faecalibacterium*, currently reclassified into the family *Oscillospiraceae* within the order *Eubacteriales*, consists of three validated species: *F. longum*, *F. butyricigenerans*, and *F. prausnitzii* [3,4]. *F. prausnitzii* is one of the predominant bacteria in the human gut, accounting for approximately 5% of the total fecal microbiota in healthy adults [5]. The relative abundance of *F. prausnitzii* in humans reveals that this species could play a crucial role in the gut microbiota, thereby impacting the physiological health of the host [6].

Recent studies have reported a correlation between low *F. prausnitzii* abundance and the risk of inflammatory bowel disease (IBD) [7,8]. In patients with Crohn’s disease (CD), a low relative population of *F. prausnitzii* represents a risk of recurrence within 6 months following treatment [9]. Likewise, in ulcerative colitis (UC) patients, there is an evident association between the *F. prausnitzii* population level and the maintenance of clinical remission [10]. Interestingly, supplementation of *F. prausnitzii* strains in vivo demonstrated anti-inflammatory effects in both the UC and CD models [11,12,13]. Moreover, *F. prausnitzii* also has a beneficial effect on improving hepatic steatosis and diabetes mellitus [14,15]. Therefore, the ingestion of *F. prausnitzii* might be a potential strategy for the treatment of human IBS and metabolic diseases. However, the health-promoting effect of *F. prausnitzii* is mostly limited to the type strain A2-165 (DSMZ 17677). Thus, there is a need to isolate more strains of *F. prausnitzii* for further functional characterization.

Although metagenomic studies have shown that *F. prausnitzii* is a predominant commensal species present in the human gut microbiome, it is difficult to isolate and culture, even under anaerobic conditions [16]. *F. prausnitzii* is an extremely oxygen-sensitive (ESO) bacterium that survives for <2 min when exposed to air [17]. As a consequence, few *F. prausnitzii* strains have been successfully isolated, resulting in the constant updating of the phylogenetic relationship among *F. prausnitzii* strains. Furthermore, a large number of studies concerning *F. prausnitzii* are performed based on metagenomic data, with scant data available on the isolation and functionality of this strain [18,19]. Therefore, the gap between microbiological and metagenomic data needs to be narrowed to better understand the beneficial effects of this species, as well as to reveal its phylogenetic profile.

The present study aimed to isolate novel *F. prausnitzii* strains from healthy donors and further characterize the biodiversity and physiological characteristics of these strains. Metabolic and physiological tests were performed to better understand the biological properties of the isolated strains, especially in the gastrointestinal tract. Moreover, phylogenetic analysis involving the newly isolated *F. prausnitzii* strains and sequences from the literature was performed to further classify their phylogroups.

## 2. Materials and Methods

### 2.1. Bacterial Strains and Growth Conditions

The reference strain A2-165 (DSM17677, Braunschweig, Germany) and newly isolated *F. prausnitzii* strains were grown in a modified M2GSC medium [20] at 37 °C in an anaerobic workstation (Electrotek 400TG workstation, Eletrotek, West Yorkshire, UK). The M2GSC medium consisted of (per 1000 mL): 5 g yeast extract, 4 g NaHCO_3_, 5 g glucose, 2 g fructose, 2 g cellobiose, 10 g casitone, 1.0 g cysteine, 0.45 g K_2_HPO_4_, 0.45 g KH_2_PO_4_, 0.9 g NaCl, 0.9 g (NH_4_)_2_SO_4_, 0.09 g MgSO_4_, 0.09 g CaCl_2_, 1 mg resazurin, and either 200 mL (for isolation) or 10 mL (for culture) of clarified rumen fluid.

### 2.2. Sample Collection and Isolation of F. prausnitzii Strains

**Figure 1:**
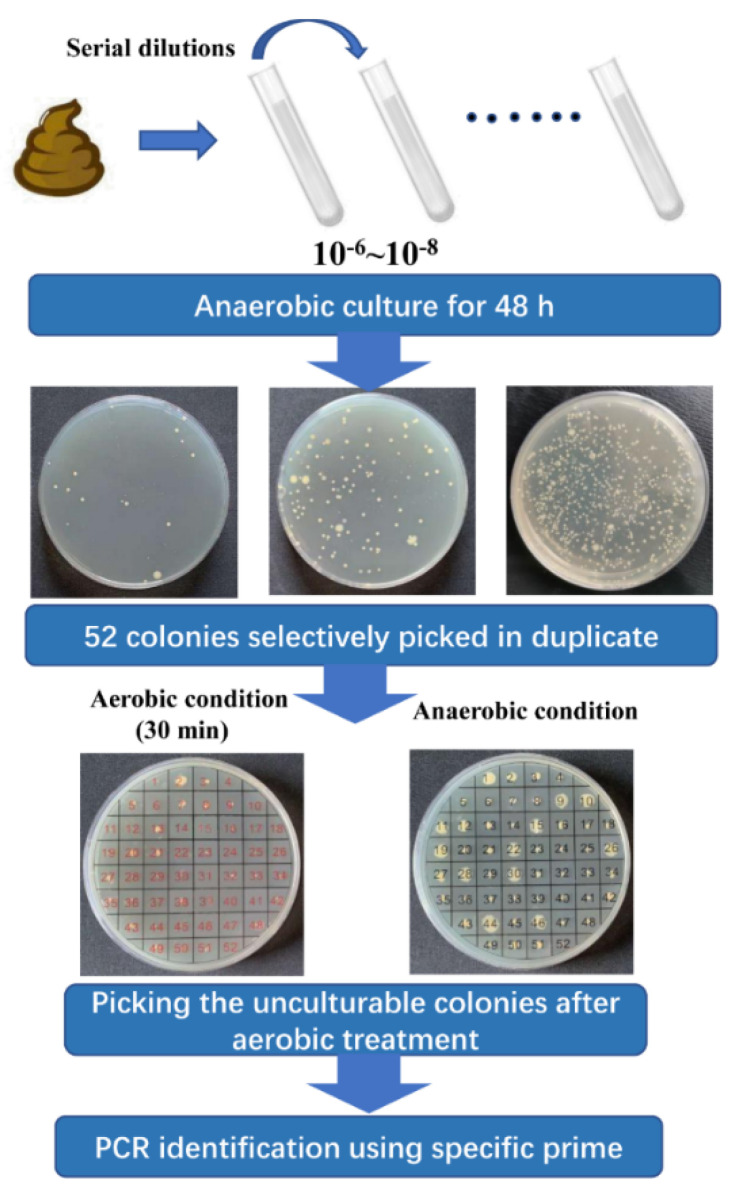
Combination of negative screening and prime-specific PCR amplification (NSPA) for isolation of *F. prausnitzii* strains from healthy human feces.

Nine healthy volunteers were recruited (Table 1) to provide fresh fecal samples, which were promptly transferred into the anaerobic workstation (N_2_:CO_2_:H_2_ = 8:1:1) within half an hour. All volunteers signed their informed consent and an agreement of confidentiality. The entire procedure for the isolation of *F. prausnitzii* strains was performed in the anaerobic workstation. The NSPA method for isolation of *F. prausnitzii* was carried out by referring to a previous study with some modifications [18]. In short, fecal samples were homogenized and serially diluted with phosphate-buffered saline (PBS) (0.1 M, pH 7.2). Dilutions 10^−6^, 10^−7^, and 10^−8^ were plated on M2GSC agar in triplicate. After 48 h of incubation, 52 varied colonies from each sample were selected and inoculated on M2GSC agar plates in duplicate and further arranged into two groups. One group was used as inoculum, while the other was treated as the negative control and exposed to the ambient air for 30 min. After 48 h of incubation, negative screening was performed to select the extremely oxygen-sensitive (EOS) strains. These EOS strains were further identified using the specific prime for *F. prausnitzii* (FPR-2F: 5′-GGAGGAAGAAGGTCTTCGG-3′; Fprau-645R: 5′-AATTCCGCCTACCTCTGCACT-3′) [5]. Finally, the isolated *F. prausnitzii* strains were stocked at -80 ℃ with 30% of glycerol. A flowchart illustrating the isolation of *F. prausnitzii* strains is shown in Figure 1.

### 2.3. Polygenetic Analysis

The colony PCR amplification of 16S rRNA from *F. prausnitzii* isolates was performed using primers 27F (5′-AGAGTTTG ATCCTGGCAG-3′) and 1492R (5′-GGCTACCTTGTTAC GACTT-3′) according to the method described by Packeiser et al. [21]. After, the 16S rRNA amplification was sequenced in Genewiz Co., Ltd. (Suzhou, China). To perform a phylogenetic analysis of the isolated strains, the 16S rRNA gene sequences for 26 *F. prausnitzii* strains were compared with those from the NCBI nucleotide database (http://www.ncbi.nlm.nih.gov, accessed on 18 October 2021). Multiple sequence alignments and a phylogenetic analysis were carried out using MEGA version 7.0 software. The phylogenetic tree based on the 16S rRNA was constructed using the maximum likelihood (ML) approach. The nucleotide sequences of the 16S rRNA reported in this paper have been deposited in the GenBank database under accession numbers OL587608—OL587633 (Table 1).

### 2.4. Scanning Electron Microscopy (SEM)

Cultures of the *F. prausnitzii* isolates were washed with PBS (0.1M, pH 7.2) and fixed with 2.5% glutaraldehyde overnight at 4 ℃. Then, the fixed specimens were dehydrated, dried, mounted, and observed using a scanning electron microscope (Hitachi SU-8010, Hitachi Ltd., Tokyo, Japan).

### 2.5. Growth Performance

To determine the growth curve of the *F. prausnitzii* isolates, the inoculum of each isolate was inoculated in 100 mL of M2GSC broth and cultured anaerobically at 37 °C for 36 h. During the culture procedure, the optical density (OD) of the culture broth was determined at λ = 600 nm using a microplate reader (Multiskan Go, Thermo Scientific, Waltham, MA, USA). Colony-forming units (CFU) for the *F. prausnitzii* isolates were determined using the Miles and Misra Method [22].

### 2.6. Resistance to pH and Bile Salts

The *F. prausnitzii* strains were inoculated in the M2GSC broth with adjusted pH levels of 3.0, 4.0, 5.0, 6.0, 7.0, 8.0, 9.0, and 10.0. The OD of the cultures was measured at λ = 600 nm using a microplate reader (Multiskan Go, Thermo Scientific, USA). Additionally, the bacterial growth in the M2GSC broth supplemented with 0.1%, 0.25%, and 0.5% (*w*/*v*) bile salts (consisting mainly of sodium glycocholate and taurocholate; Oxoid LP0055, Basingstoke, UK) was also evaluated.

### 2.7. SCFAs Analysis

The *F. prausnitzii* isolates and the reference strain A2-165 were incubated in the M2GSC broth at 37 °C for 36 h under aerobic condition. The concentrations of acetate, propionate, butyrate, and isobutyrate in the M2GSC broth before inoculation and in the *F. prausnitzii* supernatants were determined using GC–MS as previously described [23]. Each measurement for analysis was done in triplicate.

### 2.8. Determination of Antibiotics Resistance

The minimum inhibitory concentrations (MICs) of twelve antibiotics (ampicillin, vancomycin, trimethoprim, ciprofloxacin, kanamycin, gentamicin, streptomycin, neomycin, tetracycline, erythromycin, clindamycin, and chloramphenicol) were determined using the broth microdilution methods according to ISO 10932 (IDF 223:2010) [24]. *Bifidobacterium longum* ATCC 15707 was used as a quality control strain. Given the weak growth in the reference medium and EOS property of the *F. prausnitzii* isolates, the determining method based on ISO 10932 was performed with some modifications. In short, 100 uL of serial two-fold dilutions of antibiotics were added to each well of the 96 plates, followed by the addition of 100 uL diluted suspensions of the *F. prausnitzii* strains (dilution in M2GSC broth with a final concentration of approximately 3 × 10^5^). Plates were incubated in the anaerobic workstation at 37 ℃ for 36 h. MICs were recorded as the lowest concentration of an antibiotic at which visible growth was inhibited. Epidemiological cut-off (ECOFF) values were retrieved from the European Food Safety Authority (ESFA) [25].

### 2.9. Determination of Carbohydrate Utilization

In order to investigate the capabilities of carbohydrate utilization, 26 *F. prausnitzii* isolates were tested for growth on 19 different carbohydrates (glucose, maltose, fructose, arabinose, cellulose, raffinose, rhamnose, ribose, sucrose, xyloses, mannose, mannitol, galactose, lactose, trehalose, sorbose, soluble starch, inulin, and 2′-FL). The isolated strains were cultured in a modified M2GSC broth that was supplemented with a given carbohydrate (final concentration 0.5%, *w*/*v*), and the OD at 600 nm was recorded after 36 h of incubation. M2GSC broth without the addition of a carbohydrate substrate served as the negative control, while the cultures of *F. prausnitzii* strains in normal M2GSC broth were set as the positive control. The OD ratio of cultures with a given carbohydrate substrate to the positive control was used to distinguish no growth (<25% positive), moderate growth (25–75% positive), and good growth (>75% positive). All tests were done in triplicate.

### 2.10. Statistical Analysis

R (version 3.5) was used for data analysis and visualization. Results are presented as mean ± SEM. The phylogenetic tree was reconstructed using an online platform (https://www.evolgenius.info/evolview, accessed on 10 November 2021).

## 3. Results

### 3.1. Isolation and Identification of F. prausnitzii Strains

**Figure 2:**
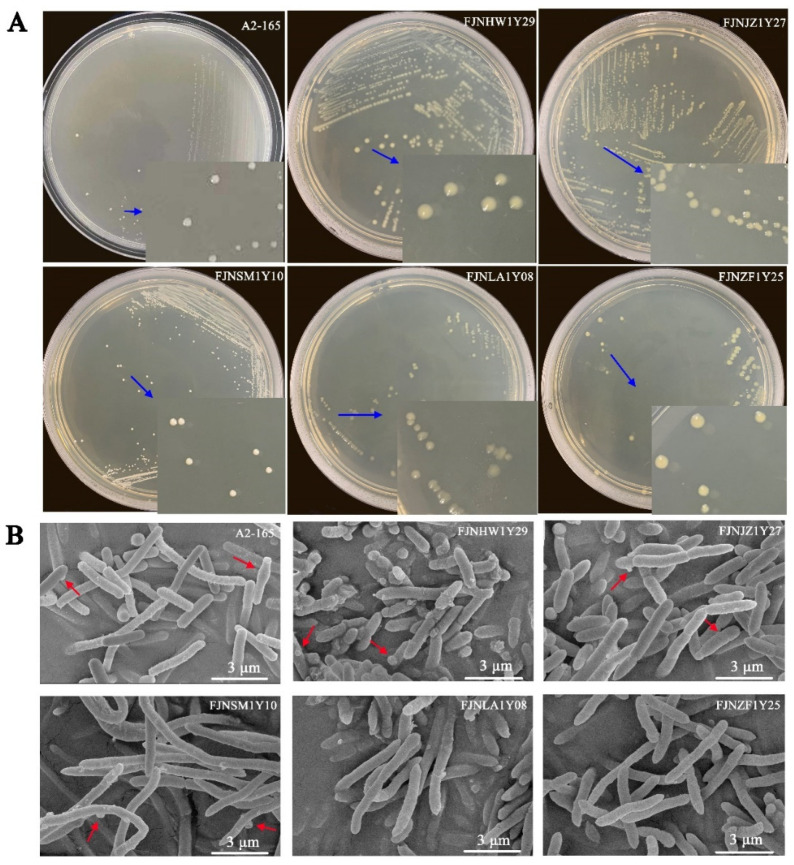
Colony and scanning electron microscopy images of *F. prausnitzii* strains: (**A**) the colony images of *F. prausnitzii* isolates and (**B**) the scanning electron microscopy images of *F. prausnitzii* isolates. The blue arrows show that colonies of *F. prausnitzii* isolates were 2–4 mm in diameter, circular, and opaque to transparent. The red arrows indicate the special ultrastructure of “swelling”. Scale bars indicate 3 μm.

Based on the NSPA method, we identified 34.6–57.7% EOS strains in the fecal bacteria of healthy donors (Table 1). These EOS isolates were further identified by *F. prausnitzii*-specific primers. Consequently, 26 *F. prausnitzii* isolates were characterized among the selected 468 colonies (5.56%). In the M2GSC agar plate, colonies were circular or irregular, opaque to transparent, and 2–4 mm in diameter (Figure 2A).

SEM analyses revealed that the *F. prausnitzii* isolates were long bacillus (2~10 μm) with rounded ends (Figure 2B). Moreover, most *F. prausnitzii* isolates had a similar morphotype with cell wall “swellings”, which had already been described in the analysis of the reference strain A2-165. However, not all *F. prausnitzii* isolates held the phenotype of “swellings”, such as the FJNLA1Y08 and FJNZF1Y25 strains, indicating that there may be a morphological diversity within the *F. prausnitzii* species.

### 3.2. Phylogenetic Diversity of Faecalibacterium prausnitzii Strains

**Figure 3:**
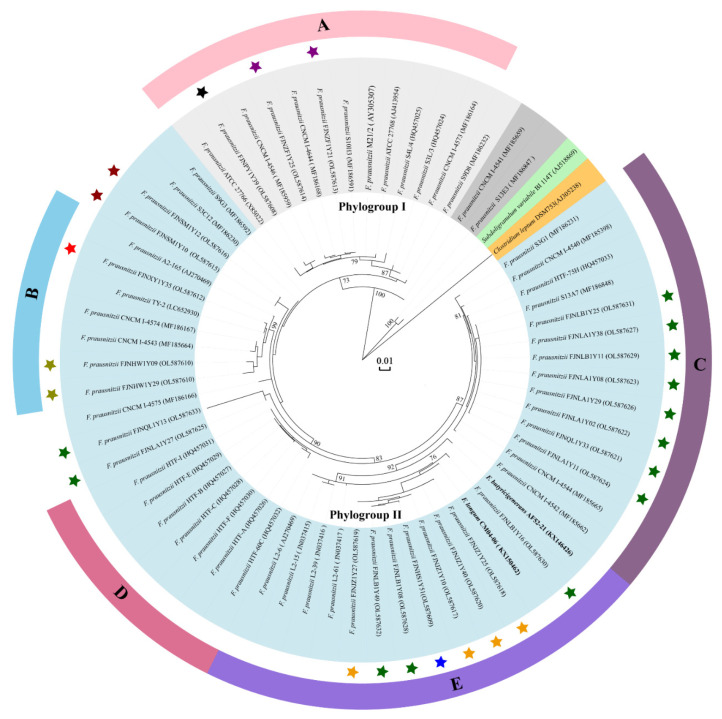
Phylogenetic tree of *F. prausnitzii* strains based on 16S rRNA gene sequences. The rooted tree was constructed with the MEGA7 software package using the maximum likelihood method. *Clostridium* *leptum* DSM 753 was used as an out-group. Bootstrap values higher than 70% are shown at the branching points. Background colors indicate different categories (light grey, phylogroup I; light blue, phylogroup II; grey, unclustered branches; light green, other genera from *oscillospiraceae* family; orange, out-group). The *F. prausnitzii* strains isolated in the present study were marked with stars (isolates derived from the same donor were presented with the same color). Colors and letters (A, B, C, D, and E) represent the tree subgroups formed by our isolated strains. The bold characters indicate type strains of two newly identified species (*F. longum* and *F. butyricigemerams*) of genus *Faecalibacterium*.

A phylogenetic tree illustrating the relationships of the 16S rRNA sequences from the *F. prausnitzii* strains is shown in Figure 3. Overall, the *F. prausnitzii* strains can be divided into two phylogroups, named phylogroup I and phylogroup II, which clearly split into five subgroups (I-A, II-B, II-C, II-D, and II-E). However, the CNCM I-4541 and S13E3 strains do not fit in any phylogroup. The S13E3 strain shares 92.88% and 93.41% 16s rRNA sequence similarities with type strains A2-165 and ATCC 27768, respectively, indicating it may not be an *F. prausnitzii* strain. Moreover, the FJNLB1Y16 strain showed a close relationship with *F. longum* CM04-06. Interestingly, several strains (FJNLB1Y25, FJNLB1Y11, FJNLB1Y16, FJNLB1Y08, and FJNLB1Y49) with distinctly different genotypes were isolated from the same donor.

### 3.3. Growth Performance of F. prausnitzii Isolates

**Figure 4:**
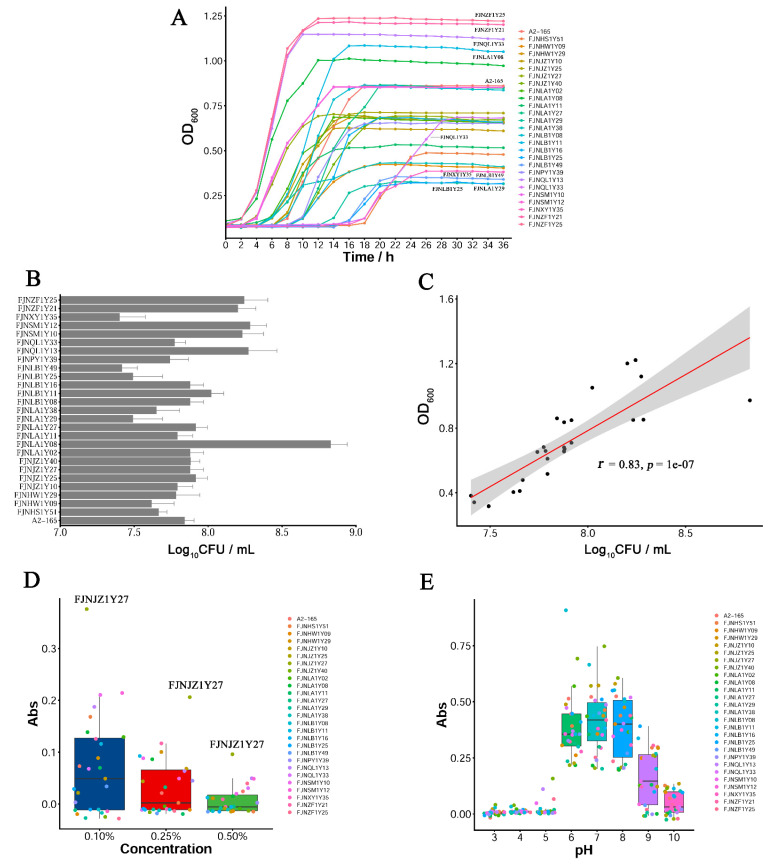
The growth performance of *F. prausnitzii* strains. (**A**) The growth curves of *F. prausnitzii* isolates; (**B**) the determination of colony-forming units (CFU) for *F. prausnitzii* strains; (**C**) the correlation of optical density with log_10_ CFU/mL; (**D**) the effect of bile salts on bacterial growth; (**E**) the effect of pH on bacterial growth.

To investigate the growth performance of the *F. prausnitzii* isolates, the growth curve, CFU counts, and resistance to pH and bile salts were evaluated. As shown in Figure 4A, the *F. prausnitzii* strains displayed different growth rates in M2GSC broth. The FJNQL1Y13, FJNLA1Y08, FJNZF1Y21, and FJNZF1Y25 strains proliferated rapidly after inoculation, reaching the stationary phase 10 h later. In contrast, the FJNLA1Y29, FJNLB1Y25, FJNLB1Y49, and FJNXY1Y35 strains exhibited a relatively flat growth curve, and their optical densities during the stationary phase were between 0.3 and 0.4, which were lower than other strains. Most strains (20/27 strains) reached the stationary phase 10 to 20 h after inoculation, except for the FJNQL1Y33 and FJNXY1Y35 strains.

To measure the number of viable *F. prausnitzii* strains in M2GSC broth, CFU counts were measured. As presented in Figure 4B, the number of viable *F. prausnitzii* strains was calculated between 2.5 × 10^7^ ~ 6.8 × 10^8^ CFU/mL in the stationary phase. There was a positive correlation between log_10_ CFU /mL and OD_600nm_ (Figure 4C, *r* = 0.83). Moreover, bile salts inhibited the growth of numerous isolates, and only a few isolates can grow in the presence of 0.1% and 0.25% (*w*/*v*) bile salts (Figure 4D). Surprisingly, the FJNJZ1Y27 isolate showed weak growth in the presence of 0.5% bile salt. The optimal pH for the growth of the *F. prausnitzii* strains was 6.0–7.0, and no growth occurred below a pH of 4.0–5.0 or above 8.0–9.0 (Figure 4E).

### 3.4. SCFAs Analysis

**Figure 5:**
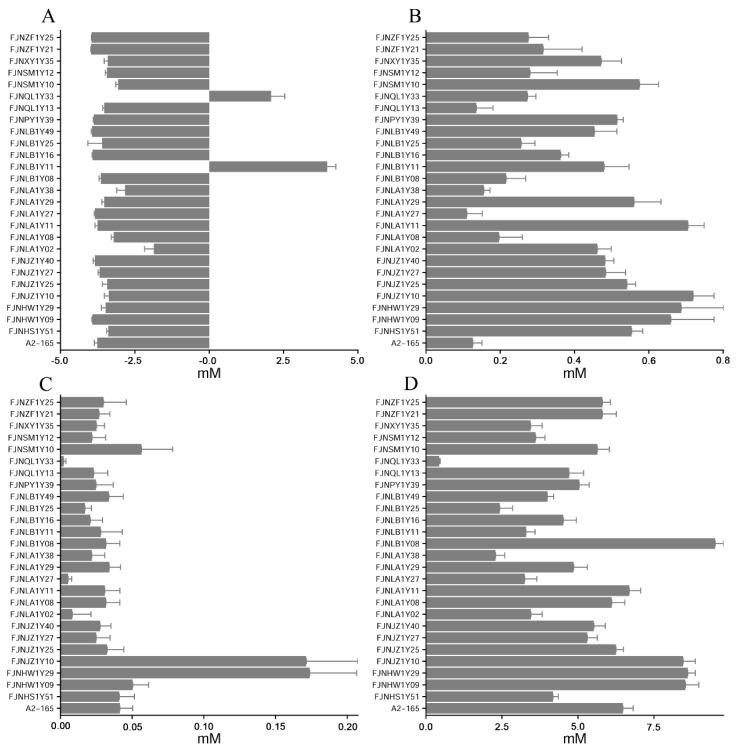
SCFAs production of *F. prausnitzii* strains in the cultural media. (**A**) Acetic acid concentration; (**B**) propionic acid concentration; (**C**) iso-butyric acid concentration; (**D**) butyric acid concentration.

The supernatant concentrations of the acetate, propionate, isobutyrate, and butyrate are shown in Figure 5. An evident reduction in acetate concentration was observed in the *F. prausnitzii* strains, except for the FJNQL1Y33 and FJNLB1Y11 isolates (Figure 5A). The low content of propionate (~0.8 mM, Figure 5B) and iso-butyrate (~0.2 Mm, Figure 5C) was observed in the fermented media of the *F. prausnitzii* isolates. The *F. prausnitzii* isolates produced butyrate ranging from 2.5 to 10 mM (Figure 5D). Moreover, the FJNLB1Y08, FJNJZ1Y10, FJNHW1Y09, and FJNHW1Y29 isolates exhibited higher butyrate-producing capability than the reference strain A2-165.

### 3.5. Resistance to Antibiotics

The MICs of different antibiotics for the *F. prausnitzii* strains are presented in Table 2. Vancomycin was the only antibiotic that all of the *F. prausnitzii* strains were susceptible to (MICs < 2 μg/mL). Besides, the *F. prausnitzii* strains tended to be susceptible to ampicillin, streptomycin, tetracycline, clindamycin, and chloramphenicol, with only <25% of resistant isolates. In contrast, most of the *F. prausnitzii* isolates were resistant to kanamycin (85.2%, 23/27 strains) and gentamicin (81.5%, 22/27 strains). For erythromycin, the resistance level and the susceptibility level were comparable, with 40.7% and 59.3% of resistant isolates, respectively. Finally, regarding ciprofloxacin, trimethoprim, and neomycin, results were varied for different isolates, with MICs ranging from 0.25 to 64 μg/mL.

### 3.6. Carbohydrate Utilization

To assess the profile of carbohydrate utilization for the *F. prausnitzii* strains, in vitro growth assays were performed involving 19 carbohydrates as sole carbon sources. As shown in Table 3, all strains displayed good growth in glucose, maltose, cellulose, and fructose. Apart from FJNPY1Y39 and FJNQL1Y33, the remaining strains were able to ferment (limited) inulin. Moreover, just two strains, FJNLA1Y27 and FJNLA1Y29, were capable of (limited) growth with arabinose as the sole carbon source; similarly, only FJNLB1Y25 was able to utilize raffinose and ribose, and just two strains, FJNZF1Y21 and FJNZF1Y25, were able to weakly grow when mannitol was utilized as the sole carbon source. None of the 27 strains were able to metabolize xylose, sorbose, and 2′-FL. The fermentation capabilities for the remaining seven carbohydrates (rhamnose, sucrose, mannose, galactose, lactose, trehalose, and soluble starch) were shown to be variable among the 27 strains (Table 3).

## 4. Discussion

*F. prausnitzii* is one of the predominant species of commensal bacteria present in the human gut [26]. Recently, it has aroused extensive attention due to its significant association with various human diseases [27,28,29]. However, the biodiversity and health-promoting effects of *F. prausnitzii* remain unclear, which could be attributed to the small number of strains isolated thus far. The NSPA method for the isolation of *F. prausnitzii* was performed based on its EOS property and prime-specific PCR amplification. Compared with the method reported by Foditsch et al. [30], two main procedures, including negative screening and prime-specific PCR amplification, were added to eliminate the non-EOS bacteria, as well as to save the cost of identification. Moreover, the NSPA method increased approximately eight-fold (52 colonies vs. 6 colonies per stool sample) the screening throughput compared to the method described by Martin et al. [18], thereby raising the isolated probability of *F. prausnitzii* per stool sample (26 isolates/9 stool samples vs. 17 isolates/13 stool samples). Here, we identified 26 *F. prausnitzii* isolates based on the NSPA method. Biodiversity and physiological characterizations, including the ultrastructural features, growth performance, SCFAs metabolism, phylogenetic diversity, resistance to antibiotics, and carbohydrate utilization, were further determined.

To date, there are three species identified in the genus *Faecalibacterium* [3]; however, phylogenetic diversity within this genus is continuously being updated with the increase in identification of new *F. prausnitzii* isolates [5,18,31,32]. Overall, the *F. prausnitzii* strains can be divided into two main phylogroups (Figure 3, phylogroups I and II) based on the analysis of 16S rRNA gene sequences, as previously described [5,18,31]; however, the assignment for certain strains was different from those reported in previous studies. The CNCM I-4575, SG93, and S3C12 strains were previously arranged into phylogroups I [18], but these three strains were clustered into phylogroup II in the present study. This discrepancy could be attributed to the alteration of the evolutional distance with the addition of the newly isolated *F. prausnitzii*. For consistency with previously used taxonomy [18,31,32], the newly observed subgroups were named as I-A, II-B, II-C, II-D, and II-E (subgroups II-D and II-E were newly defined based on the genetic distance). It should be noted, however, that subgroup II-C, containing the strains CNCM I-4540, CNCM I-4542, and CNCM I-4544, appears to have a common ancestor with phylogroup I, based on the whole genome analysis [5]; this is probably caused by inconsistent similarities of the 16S rRNA gene sequences among the *F. prausnitzii* strains with that of the whole genomes. For instance, Fitzgerald et al. described that the CNCM I-4540 strain has a higher ANI (average nucleotide identity) with ATCC 27768 (85.2%, type strain of phylogroup I) than that with A2-165 (83.6%, type strain of phylogroup II) [5], but the result is contrary in the 16S rRNA gene sequence similarities (97.5% vs. 98.1%-). Interestingly, several strains (FJNLB1Y25, FJNLB1Y11, FJNLB1Y16, FJNLB1Y08, and FJNLB1Y49) with distinct subgroups were isolated from the same donor, indicating that population diversity could be present in a single human gut microbiome. Surprisingly, Filippis et al. identified 22 different species-level genome bins through analysis of the metagenome-assembled genomes, with 12 globally spread in the human gut [19]. Furthermore, some strains, such as FJNQL1Y13, FJNLA1Y27, and S9D8, cannot be arranged into any subgroups, suggesting that the phylogenetic relationship between *F. prausnitzii* remains unknown. These results suggested that the 16S rRNA of *F. prausnitzii* exhibits a high level of diversity, indicative of a potential for separation into new species-level taxa, which should be further verified by whole-genome sequence analysis and biochemical characterization.

When examining the physiological properties of the *F. prausnitzii* isolates, it is clear that cell morphotypes, as well as the growth performance, were varied. For instance, most *F. prausnitzii* isolates displayed similar ultrastructure with cell wall “swellings”, but these morphotypes were not observed in the FJNLA1Y08 and FJNZF1Y25 isolates, indicating that the *F. prausnitzii* isolates may have morphological variation (Figure 2B). Furthermore, under anaerobic condition, most *F. prausnitzii* isolates grew fast in the M2GSC broth, reaching the stationary phase 10 to 20 h after inoculation. In contrast, the FJNQL1Y33 and FJNXY1Y35 isolates reached the stationary phase 28 h after inoculation (Figure 4A). Additionally, the number of viable *F. prausnitzii* strains in the stationary phase had a large span, ranging from 2.5 × 10^7^~6.8 × 10^8^ CFU/mL (Figure 4B). These phenotypic differences provide new information about the cultural and physiological characteristics of *F. prausnitzii*.

To imitate the adverse conditions encountered by *F. prausnitzii* in vivo, growth at a low pH or in the presence of bile salts was determined. The appropriate pH for the growth of *F. prausnitzii* ranged from 6.0 to 7.0, which was consistent with the results of a previous study [30]. The median fasting gastric pH presented in adults is approximately 1.5, which increases and peaks at approximately pH 6.5 after a standard meal, then decreases continuously returning to the fasting state value over the subsequent two hours [33]. In addition, the pH in the intestine varied between 5.0 and 8.0 [34]. Thus, a low pH of the stomach is considered a barrier to the ingestion of probiotics. The results of the present study suggested that *F. prausnitzii* could be ingested after food consumption to escape a low gastric pH, which is detrimental to bacterial cells (Figure 4F). Moreover, the *F. prausnitzii* isolates were found to be vulnerable to bile salts in vitro (Figure 4E). Previous studies have demonstrated that hepatic and gastrointestinal disorders arise along with increased luminal concentrations of bile salts, explaining the low counts of *F. prausnitzii* displayed among individuals suffering from such disorders [35,36].

SCFAs have various positive effects on gut health, such as shaping the gut environment, influencing the physiology of the colon, or being used as energy sources by the intestinal microbiota [37,38]. Moreover, SCFAs play a crucial role in energy homeostasis, insulin sensitivity, and glucose and lipid metabolism [39]. As a dominant member of the gut microbiota, the ability of *F. prausnitzii* to produce SCFAs requires investigation to evaluate the potential as a health-promoting commensal bacterium. SCFAs metabolic measurements for our *F. prausnitzii* isolates indicated that *F. prausnitzii* is an acetate consumer and butyrate producer, as previously described [18,32]. Moreover, *F. prausnitzii* can also produce minor amounts of propionate and isobutyrate (Figure 5). Importantly, the FJNLB1Y08, FJNJZ1Y10, FJNHW1Y09, and FJNHW1Y29 isolates produced higher levels of butyrate than the reference strain A2-165, showing much more potential for future probiotic candidates.

The EFSA emphasizes the need for determining whether a candidate probiotic has no acquired or transferable resistance factor before declaring it safe for human and animal consumption and obtaining qualified presumption of safety (QSP) status [40]. Furthermore, the World Health Organization and the Food and Agriculture Organization recommend that probiotics used for food consumption should be evaluated for the safety of antibiotic resistance in commercial applications [41]. However, the antimicrobial resistance profiles of the *F. prausnitzii* strains were not well characterized due to the limited number of strains isolated. Here, we tested their antimicrobial susceptibilities based on the EFSA guidance. Our findings suggested that the *F. prausnitzii* strains exhibited high-level sensitivity to vancomycin, ampicillin, clindamycin, and chloramphenicol, as well as resistance to kanamycin, as previously described [18,32]. Surprisingly, data obtained in the present study revealed that most *F. prausnitzii* isolates were gentamicin resistant (MICs ranging from 2 to 256 μg/mL) and streptomycin sensitive (MICs ranging from 0.5 to 16 μg/mL), which was contrary to the results reported in the previous study [18]. This inconsistency may be attributed to the intraspecies genomic diversity of *F. prausnitzii* strains, resulting in phenotypic changes. According to the findings of this and previous investigations, the genomics of *F. prausnitzii* strains might contain kanamycin-resistant genes, as well as the gentamicin- and streptomycin-resistant genes. Given the successful transfer of antibiotic-resistant genes from a commensal to a pathogenic bacterium [42], evaluating whether there is no transferable antibiotic resistance gene is crucial for *F. prausnitzii* strains to be granted QSP status.

The capacity to utilize carbohydrates is critical for commensal bacteria adapting to the dynamic and multiple intestinal microbiota circumstances. We thus determined the capacities of *F. prausnitzii* isolates to metabolize diverse carbohydrates. Our findings showed that all *F. prausnitzii* isolates were able to ferment a common set of sugars, including maltose, cellulose, and fructose. In contrast, all isolates were not able to utilize 2′-FL, one of the most abundant HMOs present in the human breast milk and a significant early-life stable conization factor for gut microbes [43]. The inability to metabolize 2′-FL might metabolically explain the reason why the amount of *F. prausnitzii*-specific RNA in infant stools is under the detection threshold [10], indicating that *F. prausnitzii* hardly resides in the infant gut. Additionally, almost all strains were able to utilize inulin, a dietary fiber beneficial for human health. Inulin was approved as a functional food ingredient to improve the nutritional value of food products manufactured by the Food and Drug Administration in 2018 [44]. Given its favorable impact on commensal bacteria, inulin has been used as a prebiotic for *Bifidobacterium* proliferation [45,46]. Consequently, inulin might be considered as a dietary supplement to increase the population of *F. prausnitzii* in the human gut.

This study still has some limitations that need to be mentioned. For example, the phylogenetic relationship among the *F. prausnitzii* strains was not verified by the whole genome sequence analysis. Compared with the 16S rRNA gene sequence, core genomes have higher resolution for the phylogenetic analysis of intraspecies bacteria [47]. Moreover, determining the genotype–phenotype association is also our future direction.

In summary, this study isolated 26 novel *F. prausnitzii* strains from human feces based on the NSPA method and further investigated the biodiversity and physiological characteristics of these strains. Differences in the ultrastructural feature, growth performance, SCFAs metabolism, antibiotic resistance, and carbohydrate utilization capabilities indicated a high intraspecies diversity among *F. prausnitzii* strains. Furthermore, the phenotypic characterization in combination with the phylogenetic analysis proposed a potential for *F. prausnitzii* separating into new species-level taxa. This work substantially increased the number of *F. prausnitzii* strains derived in the human gut and provided new insights into the biodiversity and physiological characteristics of *F. prausnitzii*.

## Figures and Tables

**Table 1:** Information about fecal samples and newly identified *F. prausnitzii* strains.

Subject	Sex	Age (Years)	Isolation Source	%EOS	Identified *F. prausnitzii* Strains	Accession Number
A	M	28	Human feces	42.3	FJNHS1Y51	OL587609
B	M	25	Human feces	53.5	FJNJZ1Y10	OL587617
					FJNJZ1Y25	OL587618
					FJNJZ1Y27	OL587619
					FJNJZ1Y40	OL587620
C	F	56	Human feces	34.6	FJNPY1Y39	OL587608
D	F	25	Human feces	38.5	FJNQL1Y13	OL587633
					FJNQL1Y33	OL587621
E	M	28	Human feces	57.7	FJNLA1Y02	OL587622
					FJNLA1Y08	OL587623
					FJNLA1Y11	OL587624
					FJNLA1Y27	OL587625
					FJNLA1Y29	OL587626
					FJNLA1Y38	OL587627
					FJNLB1Y08	OL587628
					FJNLB1Y11	OL587629
					FJNLB1Y16	OL587630
					FJNLB1Y25	OL587631
					FJNLB1Y49	OL587632
F	F	28	Human feces	38.5	FJNSM1Y10	OL587615
					FJNSM1Y12	OL587616
G	M	32	Human feces	40.4	FJNHW1Y09	OL587610
					FJNHW1Y29	OL587611
H	F	30	Human feces	36.5	FJNXY1Y35	OL587612
I	M	36	Human feces	51.9	FJNZF1Y21	OL587613
					FJNZF1Y25	OL587614

Note: F, female; M, male.

**Table 2:** Antimicrobial resistance profile of the *F. prausnitzii* strains isolated from healthy human feces.

Antibiotic	Distribution of Isolates														Percentage of Resistant Isolates (%)	MIC_50_	MIC_90_
	~0.03	~0.25	0.5	1	2	4	8	16	32	64	128	256	512	1024~			
Trimethoprim	-	1	-	2	-	-	-	1	3	20	-	-	-	-	ND	64	64
Ciprofloxacin	-	1	2	1	4	13	4	2	-	-	-	-	-	-	ND	4	8
Ampicillin	-	2	12	7	2	3	-	1	-	-	-	-	-	-	22.2	0.5	4
Vancomycin	-	16	7	2	2	-	-	-	--		-	-	-	-	0	0.25	1
Kanamycin	-	-	-	-	-	-	1	3	9	5	2	-	3	4	85.2	64	1024
Gentamicin	-	-	-	-	1	4	2	10	4	1	1	4	-	-	81.5	16	256
Streptomycin	-	-	2	1	4	7	11	1	-	-	1	-	-	-	7.4	4	8
Tetracycline	-	18	4	1	-	1	-	-	3	-	-	-	-	-	14.8	0.25	4
Clindamycin	10	5	3	1	1	5		2	-		-	-	-	-	7.4	0.25	4
Erythromycin	-	5	3	8	3	-	8	-	-	-	-	-	-	-	40.7	1	8
Chloramphenicol	-	5	5	4	6	1	3	3	-	-	-	-	-	-	22.2	1	8
Neomycin	-	-	-	-	-	3	11	9	1	-	3	-	-		ND	8	32

Distribution of the minimal inhibitory concentrations (MICs) for the 26 *F. prausnitzii* isolates and the reference strain A2-165 (DSM 17677) among the variable antimicrobial concentration (0.03–1024 μg/mL). Vertical bars represent the breakpoint between sensitivity and resistance. The resistance breakpoints are based on Gram+ bacterium from EFSA. The MICs of each *F. prausnitzii* strain are present in Table S1. MIC_50_, the MIC value at which at least 50% isolates were inhibited; MIC_90_, the MIC value at which at least 90% isolates were inhibited; ND, not defined.

**Table 3:** Carbohydrate utilization of *Faecalibacterium prausnitzii* strains.

Strains	Glu	Mal	Fru	Ara	Cel	Raf	Rha	Rib	Suc	Xyl	Man	Mai	Gal	Lac	Tre	Sor	SS	In	2F
A2-165	+	+	+	-	+	-	-	-	-	-	-	-	+	+	-	-	+	+	-
FJNHS1Y51	+	+	+	-	+	-	-	-	-	-	w	-	w	w	w	-	-	+	-
FJNJZ1Y10	+	+	+	-	+	-	w	-	w	-	+	-	+	w	-	-	-	+	-
FJNJZ1Y25	+	+	w	-	+	-	w	-	w	-	+	-	+	+	-	-	-	+	-
FJNJZ1Y27	+	+	+	-	+	-	w	-	w	-	w	-	+	w	+	-	-	w	-
FJNJZ1Y40	+	w	+	-	+	-	w	-	+	-	w	-	w	w	w	-	+	w	-
FJNPY1Y39	+	-	+	-	-	-	w	-	-	-	-	-	-	-	-	-	-	-	-
FJNQL1Y13	+	-	+	-	-	-	-	-	w	-	-	-	-	-	-	-	-	w	-
FJNQL1Y33	+	w	w	-	w	-	-	-	+	-	-	-	-	-	-	-	-	-	-
FJNLA1Y02	+	w	+	-	+	-	-	-	w	-	w	-	w	w	-	-	-	+	-
FJNLA1Y08	+	-	+	-	-	-	-	-	-	-	w	-	-	-	-	-	-	+	-
FJNLA1Y11	+	-	+	-	-	-	-	-	-	-	w	-	-	-	-	-	-	+	-
FJNLA1Y27	+	+	+	w	+	-	-	-	+	-	+	-	-	-	+	-	-	+	-
FJNLA1Y29	+	+	+	+	+	-	-	-	+	-	+	-	+	+	+	-	-	+	-
FJNLA1Y38	+	+	+	-	+	-	-	-	w	-	w	-	w	w	+	-	-	+	-
FJNLB1Y08	+	+	+	-	+	-	-	-	-	-	w	-	+	-	-	-	-	w	-
FJNLB1Y11	+	w	+	-	+	-	-	-	-	-	w	-	w	w	-	-	-	w	-
FJNLB1Y16	+	+	+	-	w	-	-	-	+	-	+	-	+	+	-	-	-	+	-
FJNLB1Y25	+	+	w	-	+	+	+	+	+	-	w	-	+	+	+	-	w	+	-
FJNLB1Y49	+	+	+	-	+	-	-	-	-	-	w	-	+	+	w	-	w	w	-
FJNSM1Y10	+	+	+	-	+	-	w	-	-	-	w	-	w	w	-	-	-	w	-
FJNSM1Y12	+	+	+	-	+	-	+	-	w	-	+	-	+	w	-	-	-	+	-
FJNHW1Y09	+	+	w	-	+	-	-	-	w	-	+	-	w	w	+	-	-	+	-
FJNHW1Y29	+	+	w	-	w	-	-	-	w	-	w	-	w	w	w	-	w	+	-
FJNXY1Y35	+	+	+	-	+	-	-	-	-	-	w	-	+	-	-	-	-	w	-
FJNZF1Y21	+	+	w	-	w	-	+	-	w	-	w	w	w	w	+	-	w	w	-
FJNZF1Y25	+	+	w	-	w	-	+	-	w	-	w	w	w	w	+	-	w	+	-

The *F. prausnitzii* strains were cultured in a modified M2GSC broth that was supplemented with a given carbohydrate (final concentration 0.5%, *w*/*v*), and the OD at 600 nm was recorded after 36 h of incubation. The cultures of the *F. prausnitzii* strains in normal M2GSC broth were set as the positive control. Note: +, >75% positive (good growth); -, 0–25% positive (no growth); w, 25–75% positive (moderate growth). Glu, glucose; Mal, maltose; Fru, fructose; Ara, arabinose; Cel, cellulose; Raf, raffinose; Rha, rhamnose; Rib, ribose; Suc, sucrose; Xyl, xylose; Man, mannose; Mai, mannitol; Gal, galactose; Lac, lactose; Tre, trehalose; Sor, sorbose; SS, soluble starch; In, inulin; 2F, 2′-FL.

## Data Availability

The accession numbers for all 16S rRNA gene sequences used in this study are presented in Table 1.

## Supplementary Materials

The following supporting information can be downloaded at: https://www.mdpi.com/article/10.3390/microorganisms10020297/s1, Table S1: Minimum inhibitory concentrations (MIC) (μg/mL) of tested antibiotics for each *F. prausnitzii* isolates and reference strain A2-165.
