# Supplementary material for: Biodiversity and Physiological Characteristics of Novel Faecalibacterium prausnitzii Strains Isolated from Human Feces
Source: Microorganisms. 2022 Jan 26;10(2):297. doi: 10.3390/microorganisms10020297 (PMC8876097; doi:10.3390/microorganisms10020297)
Supplement: Supplementary file 1 [file microorganisms-10-00297-s001.zip › microorganisms-1527788-supplementary.pdf]

Supplementary data

Table S1. Minimum inhibitory concentrations (MIC) (μg/mL) of tested antibiotics for each of the *F. prausnitzii* isolates and reference strain A2-165.

Strins	TRI	CIP	AMP	VAN	KAN	GEN	STR	TET	CLI	ERY	CHL	NEO
	ND	ND	**1**	**4**	**16**	**4**	**8**	**2**	**4**	**1**	**4**	ND
A2-165	32	4	1(S)	0.5(S)	128(R)	32(R)	16(R)	0.25(S)	0.5(S)	0.5(S)	0.5(S)	16
FJNHS1Y51	1	0.25	4(R)	0.5(S)	64(R)	16(R)	8(S)	0.5(S)	4(S)	2(R)	8(R)	8
FJNHW1Y09	64	8	0.5(S)	<0.25(S)	8(S)	2(S)	0.5(S)	0.25(S)	<0.03(S)	8(R)	2(S)	8
FJNHW1Y29	32	4	0.25(S)	0.5(S)	16(S)	4(S)	0.5(S)	0.25(S)	<0.03(S)	0.25(S)	1(S)	8
FJNJZ1Y10	64	4	0.5(S)	<0.25(S)	32(R)	16(R)	4(S)	0.25(S)	<0.03(S)	1(S)	<0.125(S)	16
FJNJZ1Y25	64	4	0.5(S)	<0.25(S)	32(R)	16(R)	4(S)	<0.125(S)	<0.03(S)	1(S)	<0.125(S)	16
FJNJZ1Y27	64	4	0.5(S)	<0.25(S)	32(R)	16(R)	4(S)	<0.125(S)	<0.03(S)	1(S)	16(R)	16
FJNJZ1Y40	64	4	1(S)	<0.25(S)	32(R)	16(R)	4(S)	0.25(S)	<0.03(S)	1(S)	<0.125(S)	16
FJNLA1Y02	0.25	2	1(S)	<0.25(S)	32(R)	16(R)	8(S)	<0.125(S)	4(S)	1(S)	2(S)	16
FJNLA1Y08	64	0.5	0.5(S)	2(S)	>1024(R)	256(R)	8(S)	32(R)	2(S)	0.125(S)	8(R)	128
FJNLA1Y11	1	4	2(R)	1(S)	32(R)	16(R)	8(S)	<0.125(S)	4(S)	1(S)	1(S)	8
FJNLA1Y27	64	8	1(S)	<0.25(S)	16(S)	4(S)	8(S)	<0.125(S)	0.5(S)	8(R)	16(R)	4
FJNLA1Y29	64	4	0.5(S)	<0.25(S)	32(R)	16(R)	2(S)	<0.125(S)	<0.03(S)	2(R)	<0.125(S)	8
FJNLA1Y38	16	2	2(R)	0.5(S)	512(R)	32(R)	4(S)	0.25(S)	4(S)	1(S)	16(R)	8
FJNLB1Y08	32	4	0.5(S)	<0.25(S)	32(R)	4(S)	1(S)	0.25(S)	0.25(S)	0.125(S)	0.5(S)	4
FJNLB1Y11	64	2	4(R)	<0.25(S)	512(R)	128(R)	8(S)	1(S)	1(S)	2(R)	2(S)	128
FJNLB1Y16	64	8	4(R)	<0.25(S)	64(R)	16(R)	4(S)	<0.125(S)	<0.03(S)	8(R)	0.25(S)	16
FJNLB1Y25	64	2	0.5(S)	0.5(S)	64(R)	8(R)	8(S)	0.25(S)	0.125(S)	0.25(S)	4(S)	8
FJNLB1Y49	64	4	1(S)	<0.25(S)	64(R)	16(R)	8(S)	0.25(S)	0.25(S)	0.5(S)	2(S)	8
FJNPY1Y39	64	4	0.5(S)	<0.25(S)	128(R)	64(R)	8(S)	0.25(S)	0.06(S)	0.5(S)	0.5(S)	8
FJNQL1Y13	64	0.5	0.125(S)	2(S)	512(R)	256(R)	128(R)	32(R)	16(R)	8(R)	8(R)	128
FJNQL1Y33	64	1	16(R)	1(S)	64(R)	32(R)	8(S)	4(R)	0.5(S)	0.125(S)	2(S)	16
FJNSM1Y10	64	8	1(S)	<0.25(S)	16(S)	4(S)	2(S)	0.5(S)	<0.03(S)	8(R)	0.5(S)	4
FJNSM1Y12	64	4	1(S)	<0.25(S)	32(R)	8(R)	8(S)	0.25(S)	4(S)	8(R)	1(S)	8
FJNXY1Y35	64	16	0.5(S)	0.5(S)	>1024(R)	256(R)	2(S)	0.5(S)	16(R)	8(R)	2(S)	32
FJNZF1Y21	64	4	0.5(S)	<0.25(S)	>1024(R)	32(R)	4(S)	32(R)	<0.03(S)	1(S)	0.5(S)	16
FJNZF1Y25	64	16	0.5(S)	0.5(S)	>1024(R)	256(R)	2(S)	0.5(S)	0.25(S)	8(R)	1(S)	8

Notes: TRI, trimethoprim; CIP, ciprofloxacin; AMP, ampicillin; VAN, vancomycin; KAN, kanamycin; GEN, gentamicin; STR, streptomycin; TET, tetracycline; CLI, clindamycin; ERY, erythromycin; CHL, chloramphenicol; NEO, neomycin. Numbers in bold represent the breakpoint (BP) based on Gram+ bacterium from EFSA. R, resistance (> BP). S, sensitivity (≤ BP). ND, not defined.
